# Supplementary material for: CD274 (PD-L1) Polymorphisms as Predictors of Efficacy in First-Line Platinum-Based Chemotherapy for Extensive-Stage Small Cell Lung Cancer
Source: Int J Mol Sci. 2025 Apr 29;26(9):4245. doi: 10.3390/ijms26094245 (PMC12072405; doi:10.3390/ijms26094245)
Supplement: Supplementary file 1 [file ijms-26-04245-s001.zip › ijms-3566919-supplementary.pdf]

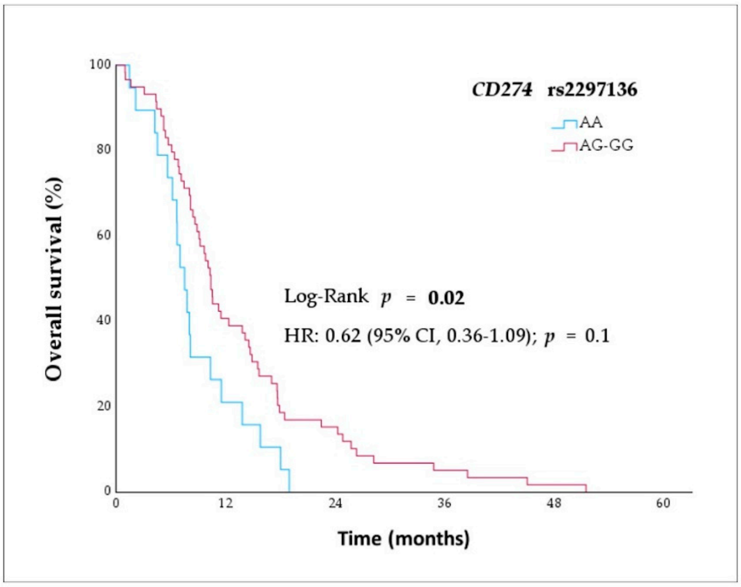

Supplementary Figure S1. Overall survival curves of extensive stage-small cell lung cancer patients according to the *CD274* rs2297136 variant in a dominant model of inheritance.

Supplementary Table S1. Analyses between the immune checkpoint genetic variants and progression-free survival of extensive stage-small cell lung cancer patients

		**Progression-free survival**				
		**Univariate analysis**			**Multivariate analysis**	
**SNP**	**n = 75**	**mPFS (95%CI), months**	**HR (95% CI)**	**[a]*p*-value**	**HR (95% CI)**	**[b]*p*-value**
CD274 rs4143815						
GG	37	6.4 (5.9-6.8)	Reference (1)	0.06		
GC	31	5.3 (4.2-6.5)	1.83 (1.09-3.06)			
CC	7	5.5 (4.9-6.1)	1.08 (0.48-2.45)			
GC-CC	38	5.3 (4.9-5.7)	1.60 (0.99-2.57)	0.05	1.33 (0.80-2.20)	0.27
CD274 rs2297136						
AA	18	4.6 (3.8-5.3)	Reference (1)	**0.007**		
AG	43	6.1 (5.2-6.9)	0.58 (0.33-1.02)			
GG	14	6.8 (6.2-7.4)	0.30 (0.14-0.66)			
AG-GG	57	6.3 (5.7-6.8)	0.50 (0.29-0.87)	**0.01**	0.52 (0.29-0.93)	**0.03**
CD274 rs2282055						
TT	42	6.1 (5.1-7.0)	Reference (1)	**0.001**		
TG	28	6.1 (4.4-7.8)	0.79 (0.48-1.28)			
GG	5	4.6 (0.0-9.7)	4.48 (1.64-12.23)			
TT-TG*	70	6.1 (5.6-6.6)	0.20 (0.08-0.55)	**<0.001**	0.23 (0.09-0.64)	**0.005**
CD274 rs822336						
GG	21	4.8 (3.9-5.8)	Reference (1)	**0.02**		

GC	35	6.5 (5.9-7.0)	0.48 (0.27-0.84)			
CC	19	6.6 (5.9-7.3)	0.50 (0.26-0.96)			
GC-CC	54	6.5 (6.0-7.0)	0.48 (0.29-0.82)	**0.006**	0.41 (0.23-0.73)	**0.002**
PDCD1 rs2227981						
GG	27	6.5 (3.7-9.2)	Reference (1)	0.66		
GA	35	5.9 (4.8-6.9)	1.26 (0.75-2.11)			
AA	13	6.1 (5.2-7.1)	1.22 (0.63-2.40)			
PDCD1 rs10204525						
CC	65	6.1 (5.2-7.0)	Reference (1)	0.69		
CT	10	5.5 (4.3-6.7)	0.87 (0.45-1.71)			
PDCD1 rs11568821						
CC	60	6.1 (5.1-7.0)	Reference (1)	0.26		
CT	13	6.1 (4.1-8.1)	1.19 (0.65-2.18)			
TT	2	4.4 (NA-NA)	3.08 (0.72-13.10)			
PDCD1 rs7421861						
AA	31	5.4 (4.4-6.4)	Reference (1)	0.30		
AG	37	6.5 (5.9-7.1)	1.01 (0.61-1.67)			
GG	7	4.4 (4.2-4.6)	1.87 (0.81-4.33)			

Supplementary Table S1. Analyses between the immune checkpoint genetic variants and progression-free survival of extensive stage-small cell lung cancer patients (**Continuation**)

		**Progression-free survival**				
		**Univariate analysis**			**Multivariate analysis**	
**SNP**	**n = 75**	**mPFS (95%CI), months**	**HR (95% CI)**	**[a]*p*-value**	**HR (95% CI)**	**[b]*p*-value**
CTLA4 rs4553808						
AA	47	6.1 (5.1-7.1)	Reference (1)	0.82		
AG	26	5.9 (4.4-7.4)	1.17 (0.71-1.90)			
GG	2	6.1 (NA-NA)	0.93 (0.22-3.86)			
CTLA4 rs231775						
AA	34	6.1 (5.9-6.3)	Reference (1)	**0.005**		
AG	32	6.3 (4.6-7.9)	0.94 (0.58-1.55)			
GG	9	4.6 (3.9-5.3)	3.10 (1.43-6.72)			
AA-AG*	66	6.1 (5.7-6.5)	0.31 (0.15.0.066)	**0.001**	0.30 (0.14-0.63)	**0.002**
CTLA4 rs3087243						
GG	22	4.8 (3.8-5.7)	Reference (1)	**0.046**		
GA	36	6.1 (5.6-6.5)	0.50 (0.29-0.89)			
AA	17	6.8 (4.7-8.9)	0.56 (0.29-1.06)			
GA-AA	53	6.2 (5.7-6.6)	0.52 (0.31-0.89)	**0.01**	0.602 (0.35-1.03)	0.07
LAG3 rs2365095						
CC	36	6.5 (5.9-7.0)	Reference (1)	0.37		

CT	34	5.4 (4.4-6.3)	1.07 (0.66-1.75)			
TT	5	4.6 (4.1-5.0)	1.96 (0.76-5.06)			
LAG3 rs870849						
CC	31	5.5 (4.4-6.6)	Reference (1)	0.82		
CT	31	6.1 (5.2-7.0)	0.94 (0.56-1.57)			
TT	13	6.1 (3.1-9.1)	1.17 (0.60-2.25)			
LAG3 rs3782735						
AA	22	6.3 (5.0-7.5)	Reference (1)	**0.04**		
AG	43	6.1 (5.1-7.0)	1.54 (0.89-2.66)			
GG	10	4.4 (3.2-5.5)	2.84 (1.25-6.47)			
AA-AG*	65	6.1 (5.7-6.5)	0.47 (0.23-0.97)	**0.04**	0.52 (0.24-1.09)	0.08

*Reference (1) is the homozygous recessive genotype. SNP, Single-nucleotide polymorphism; mPFS, median progression-free survival; HR, hazard ratio; [a]*p* value from a long-rank test; [b]*p* value from a Cox proportional hazards model. Statistically significant *p*-values are marked in bold.

Supplementary Table S2. Univariate analyses between the immune checkpoint genetic variants and overall survival of extensive stage-small cell lung cancer patients

		**Overall survival**		
		**Univariate analysis**		
**SNP**	**n = 78**	**mOS (95%CI), months**	**HR (95% CI)**	**[a]*p*-value**
CD274 rs4143815				
GG	39	10.6 (8.0-13.2)	Reference (1)	0.42
GC	32	8.7 (6.4-10.9)	1.34 (0.83-2.16)	
CC	7	8.9 (5.4-12.4)	1.41 (0.62-3.18)	
GC-CC	39	8.9 (6.9-10.9)	1.35 (0.86-2.13)	0.19
CD274 rs2297136				
AA	19	7.5 (6.0-9.0)	Reference (1)	**0.008**
AG	45	9.8 (8.0-11.7)	0.64 (0.37-1.10)	
GG	14	11.2 (0.0-23.1)	0.31 (0.15-0.66)	
AG-GG	59	10.4 (9.4-11.4)	0.54 (0.32-0.92)	**0.02**
CD274 rs2282055				
TT	45	10.4 (9.2-11.6)	Reference (1)	0.80
TG	28	8.7 (6.5-10.8)	0.86 (0.52-1.40)	
GG	5	7.1 (3.0-11.3)	1.05 (0.40-2.74)	
TT-TG*	73	10.1 (8.7-11.5)	0.89 (0.35-2.25)	0.80
CD274 rs822336				
GG	21	7.8 (6.5-9.1)	Reference (1)	0.56
GC	38	10.3 (7.7-12.9)	0.75 (0.44-1.30)	
CC	19	10.6 (8.5-12.7)	0.76 (0.40-1.43)	
GC-CC	57	10.4 (8.9-11.9)	0.76 (0.45-1.26)	0.28
PDCD1 rs2227981				
GG	28	10.4 (7.3-13.5)	Reference (1)	0.10
GA	37	7.5 (6.0-9.0)	1.63 (0.98-2.69)	
AA	13	10.6 (9.5-11.6)	0.98 (0.50-1.90)	
PDCD1 rs10204525				
CC	67	9.7 (7.5-11.9)	Reference (1)	0.64
CT	11	9.8 (3.0-16.6)	0.86 (0.45-1.63)	
PDCD1 rs11568821				
CC	62	10.4 (8.7-12.0)	Reference (1)	0.32
CT	14	8.4 (6.4-10.4)	1.31 (0.73-2.37)	
TT	2	7.4 (NA-NA)	2.49 (0.59-10.55)	
PDCD1 rs7421861				
AA	33	10.4 (7.7-13.0)	Reference (1)	0.31
AG	38	9.7 (8.2-11.2)	1.29 (0.79-2.12)	
GG	7	8.0 (6.5-9.5)	1.81 (0.79-4.18)	

Supplementary Table S2. Univariate analyses between the immune checkpoint genetic variants and overall survival (**Continuation**)

		**Overall survival**		
		**Univariate analysis**		
**SNP**	**n = 78**	**mOS (95%CI), months**	**HR (95% CI)**	**^{a}p-value**
CTLA4 rs4553808				
AA	50	10.4 (8.7-12.1)	Reference (1)	0.79
AG	26	8.4 (6.2-10.6)	1.18 (0.73-1.92)	
GG	2	8.0 (NA-NA)	1.04 (0.25-4.28)	
CTLA4 rs231775				
AA	37	8.1 (6.8-9.5)	Reference (1)	0.45
AG	32	10.4 (8.0-12.8)	0.75 (0.46-1.22)	
GG	9	10.4 (10.3-10.5)	0.74 (0.35-1.54)	
AA-AG*	69	9.2 (7.0-11.5)	1.17 (0.58-2.36)	0.66
CTLA4 rs3087243				
GG	22	9.8 (7.6-12.0)	Reference (1)	0.27
GA	36	10.4 (7.0-13.8)	0.81 (0.47-1.39)	
AA	20	8.0 (6.7-9.4)	1.29 (0.70-2.36)	
GA-AA	56	9.2 (6.5-11.9)	0.94 (0.57-1.55)	0.81
LAG3 rs2365095				
CC	37	10.4 (7.7-13.1)	Reference (1)	0.84
CT	36	8.2 (6.0-10.4)	1.15 (0.72-1.83)	
TT	5	11.5 (6.4-16.6)	1.04 (0.41-2.66)	
LAG3 rs870849				
CC	32	8.9 (5.7-12.1)	Reference (1)	0.19
CT	32	10.6 (8.6-12.6)	0.86 (0.52-1.42)	
TT	14	8.0 (6.0-10.1)	1.55 (0.82-2.95)	
LAG3 rs3782735				
AA	23	8.2 (5.2-11.1)	Reference (1)	**0.02**
AG	44	10.4 (8.8-12.0)	0.89 (0.53-1.48)	
GG	11	5.7 (2.8-8.6)	2.33 (1.10-4.95)	
AA-AG*	67	10.3 (8.9-11.6)	0.40 (0.20-0.77)	**0.005**

*Reference (1) is the homozygous recessive genotype. SNP, Single-nucleotide polymorphism; mPFS, median progression-free survival; HR, hazard ratio; ^{a}p value from a long-rank test. Statistically significant *p*-values are marked in bold.

Supplementary Table S3. *CD274* haplotype analyses (rs2297136│rs2282055│rs822336) for progression-free survival and overall survival

		Progression-free survival				Overall survival			
		Univariate analysis		Multivariate analysis		Univariate analysis		Multivariate analysis	
**Haplotype**	**Frequence**	**BETA**	***p*-value**	**BETA**	***p*-value**	**BETA**	***p*-value**	**BETA**	***p*-value**
GTC	0.39	1.37	0.25			1.75	0.3		
ATC	0.1	-0.86	0.62			-1.66	0.5		
GGG	0.06	6.35	**0.009**	5.74	**0.01**	11.8	**<0.001**	10.7	**<0.001**
AGG	0.18	-1.26	0.39			-2.48	0.23		
GTG	0.02	5.12	0.29			14.6	**0.03**	10.3	0.09
ATG	0.25	-2.68	0.06	-2.17	0.11	-4.12	**0.04**	-3.42	0.06

Supplementary Table S4. Analyses between platinum sensitivity and the rs2297136, rs2282055 and rs822336 variants in the *CD274* gene, and the *CTLA4* rs231775 variant

			**Univariate analysis**	**Multivariate analysis**	
**SNP**	**n = 74**	**Platinum-sensitive n (%)**	***p*-value**	**OR (95% CI)**	***p*-value**
CD274 rs2297136					
AA	18	2 (11.1)	**0.002**		
AG	42	15 (35.7)			
GG	14	10 (71.4)			
AG-GG**	56	25 (44.6)	**0.01**	0.13 (0.02-0.70)	**0.02**
CD274 rs2282055					
TT	41	15 (36.6)	0.19		
TG	28	12 (42.9)			
GG	5	0 (0.0)			
TT-TG*	69	27 (39.1)	0.15	NA (NA-NA)	0.99
CD274 rs822336					
GG	21	2 (9.5)	**0.008**		
GC	34	15 (44.1)			
CC	19	10 (52.6)			
GC-CC**	53	25 (47.2)	**0.003**	0.08 (0.01-0.46)	**0.005**
CTLA4 rs231775					
AA	34	14 (41.2)	0.05		
AG	31	13 (41.9)			
GG	9	0 (0)			
AA-AG*	65	27 (41.5)	**0.02**	NA (NA-NA)	0.99

*Reference (1) is the homozygous recessive genotype. **Reference (1) is the homozygous dominant genotype. SNP, Single-nucleotide polymorphism; OR, odds ratio; NA: not available. Statistically significant p-values are marked in bold.

Supplementary Table S5. Linkage disequilibrium data for *CD274* gene variants in European populations (1000 Genomes Project Phase 3)

Variant 1	Location	Variant 2	Location	r2	D'
rs822336	5448690	rs4143815	5468257	0.12	0.57
rs2282055	5455732	rs4143815	5468257	0.14	0.38
rs2297136	5467955	rs4143815	5468257	0.14	0.59
rs822336	5448690	rs2297136	5467955	0.21	0.47
rs822336	5448690	rs2282055	5455732	0.31	0.90
